# Introducing bimetallic MOF-based electrochemical sensor for voltametric nanogram determination of sulfadimidine: various applications and a comprehensive sustainability assessment

**DOI:** 10.1186/s13065-025-01465-7

**Published:** 2025-04-18

**Authors:** Hind A. Abdullatif, Mohammed Abdelkawy, Shereen A. Boltia, Nesma M. Fahmy, Maha Kamal

**Affiliations:** 1 Pharmaceutical Chemistry Dept, Faculty of Pharmacy, Ahram Canadian University, Cairo, Egypt; 2 Analytical Chemistry Department, Faculty of Pharmacy, Cairo University, Giza, Egypt

**Keywords:** Bimetallic metal organic framework, Sulfadimidine, Voltammetry, Carbon paste electrode, Trimesic acid

## Abstract

Due to its characteristics and allowable use, the sulphonamide group remains a first-choice treatment for veterinarians when managing multiple veterinary diseases. Unfortunately, long-term consumption of food containing sulphonamide residues can result in harmful effects, especially that sulphonamides are classified in category D. Metal–organic frameworks (MOFs) have demonstrated outstanding selectivity in detecting target components due to their large surface areas and intricate structures. In this study, we present a novel voltammetric approach for determining sulfadimidine (SLD) in veterinary formulations, animal plasma, and animal-derived products, including milk and eggs. We employed a bimetallic Cu/Ni-MOF to modify a carbon paste electrode, utilizing differential pulse voltammetry (DPV) for SLD detection. The morphology of the Cu/Ni-MOF was analyzed to ensure optimal structural characteristics, and experimental conditions were optimized to achieve the best performance. A major advantage of this method is its wide linearity range (100 nM to 100,000 nM) and the ability to detect SLD at nanogram levels, with a LOD of 20 nM and a LOQ of 60 nM. These characteristics demonstrate the fabricated Cu/Ni-MOF’s capability to detect SLD at levels below its maximum residue limit (MRL) in plasma, milk, and eggs. Furthermore, the environmental impact of this method was assessed using the RGB 12 metric and compared against the AGREE, Complex GAPI, and BAGI metrics, offering a comprehensive evaluation of its analytical performance and practical advantages. This approach holds promise for curbing antibiotic misuse by providing a straightforward and effective method for SLD detection across multiple matrices.

**Supplementary Information:**

The online version contains supplementary material available at 10.1186/s13065-025-01465-7.

## Introduction

As a result of the growing demand for livestock production, a considerable number of veterinary medications is given to maintain and enhance animal health. Considered the most widely used medication in animal husbandry, antibiotics are given either therapeutically to cure infectious diseases, prophylactically to shield animals from common illnesses, or used non-therapeutically to promote growth in animals [1]. Precisely, veterinary antimicrobials are extensively used to treat dairy cattle diseases gastrointestinal, mastitis, and urinary infections [2].

Numerous investigations have shown the existence of measurable levels of antibiotic residues in animal plasma and animal products, including milk and eggs [3]. These residues are a consequence of the excessive and inappropriate use of antimicrobials in relation to dosage and frequency in animal husbandry techniques [4]. Therefore, global authorities and governmental organizations have set maximum residue limits (MRLs) for veterinary antimicrobials in animal origin foods to control human exposure and guarantee the safety of food for humans [5].

**Fig. 1:**
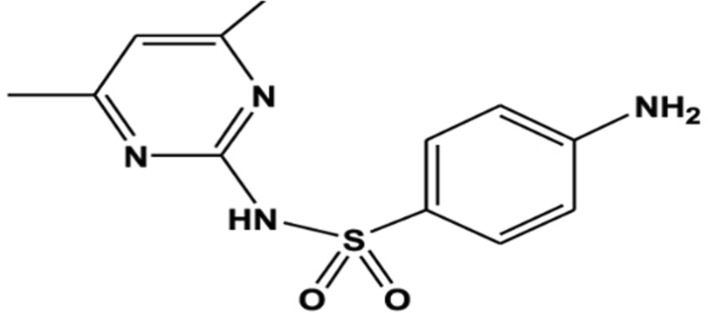
Chemical Structure of SLD

Sulfonamides are wide-spectrum antibiotics containing sulfonamide groups and amine groups. The antibacterial agent Sulfadimidine (SLD), having IUPAC name of 4-amino-N-(4,6-dimethylpyrimidin- 2-yl) benzene sulfonamide, is commonly utilized to prevent and treat urinary tract infections and other bacterial invasions, such as actinomyces [6]. According to the National Veterinary Drug Screening Program in China, Sulfonamide residues have been found in 11.5% of foods obtained from animals due to their drug-resistant toxicity [7, 8]. Sulfadimidine is responsible for 95% of the negative effects of some sulfonamides, including risks of hematopoietic damage and cancer [9]. Thus, in many nations, strict regulatory boundaries and instrumental analytical requirements have been set [10]. Ironically, Sulfonamides are widely used and frequently employed because of their cost-effectiveness, convenience of administration, and stable chemical characteristics [11]. Thus, in many nations, strict regulatory boundaries and instrumental analytical requirements have been set. Because of their cost-effectiveness, convenience of administration, and stable chemical characteristics, they are frequently employed to treat or prevent animal diseases. Several methods of determination were mentioned in literature, including spectrophotometric methods [12, 13], chromatographic methods [14–16], and electrochemical methods [17]. The drug is mentioned in the British Pharmacopoeia and assayed by potentiometric nitrite titration [18], and structure is shown in Fig. 1. The maximum residue limits (MRLs) are 100 µg/L in plasma, 25µg/L in milk, while there is no MRL specified for eggs, indicating that SLD should not be administered to laying hens, as any residue is considered unacceptable [19, 20].

Electrochemical sensors are devices that detect and measure chemical substances by converting chemical reactions into electrical signals. Electrode surface modification has a great impact on performance enhancement, particularly selectivity and sensitivity. This makes electrochemical sensors have exceptional attributes, such as being easy-to-operate, economical, sensitive, portable, and simple-to-construct. As a result, they have endless applications, quick advancements, and ongoing continuous improvement [21]. Cyclic voltammetry (CV) is an electrochemical technique used to investigate the redox behavior of chemical species. In CV, a triangular potential is applied to a working electrode with small dimensions (typically a disk of millimeter-scale diameter), and the resulting current is measured. This working electrode is part of a three-electrode system that allows current to pass through a counter electrode while the potential is precisely controlled relative to a reference electrode using a potentiostat [17]. Differential pulse voltammetry (DPV) is a widely used pulsed voltammetric technique in electroanalytical applications. In DPV, a staircase potential ramp is applied, overlaid with small, constant potential pulses. The current response is determined by subtracting the current measured at the end of the pulse (Δt2) from that just before the pulse (Δt1) [18].

The creation of 1-, 2-, and 3-dimensional (3D) molecular organic–inorganic hybrid molecules has advanced significantly in recent years. Molecular organic–inorganic hybrid compounds are generally constructed with metal ions as inorganic connectors, and multifunctional organic ligands as the linkers, to generate versatile and infinite structures, which can also be called coordination polymers [22]. Coordination polymers can be formed using ionic attraction (metal–organic frameworks, MOFs. Those MOFs are infinite crystalline networks formed when polyfunctional organic molecules bind to metal ions, which serve as coordination centers [23]. Metal–organic frameworks have become a novel class of porous materials since they have their remarkable ability to solve important worldwide issues (e.g., detecting heavy metals, and serve as energy storage devices like batteries) due to their redox- active properties [24]. MOFs have exceptionally high surface areas, which enhance the active surface available for electrochemical reactions, leading to increased sensitivity in voltametric measurements. Many MOFs contain metal nodes that act as catalytic sites, improving the efficiency of redox reactions in voltametric studies. Moreover, the tunable pore size of MOFs allows for selective adsorption of target analytes, improving specificity in electrochemical sensing applications [25]. Although MOFs are often poor conductors, incorporating conductive elements like metal nanoparticles or carbon-based materials significantly enhances electron transfer, making them suitable for electrochemical applications [26].

The most crucial factors in obtaining optimum MOFs are the chemical structure of the organic linker, and the inorganic metal ions'coordination modes [27]. The almost infinite possibilities of organic clusters ligands that can be combined with hundreds of inorganic clusters through intuitive or advanced complex assembly strategies led to the report of almost 100, 000 MOFs [28].The geometrical forms of MOFs can also be effectively determined by other factors, including the sizes of the linkers and the interacting metal ions, the technique used, and the preparation medium conditions of pH and solvent polarity [29]. Also, the functional groups on the organic linker, such as carboxylic acids, thiols, and amines, can play a further role in post-modification, pore sizes and surface area tuning of MOFs [30]. MOFs with multiple open pores, high overall porosity, and easy access to active is a particularly promising class of electrode materials to achieve better electrocatalyst performance [31]. Consequently, MOFs are nowadays implemented in extraordinary applications in diverse fields like gas storage and separation [32], sensing [33], proton conduction [34], drug delivery [35], and catalysis [28, 36].

Several strategies emerged to increase the catalytic activity, which includes optimization of coordination structures, element doping [37], or controlling the number of MOFs'available active sites using various techniques [38, 39]. Moreover, an alternative route for developing electrode materials and electrocatalysts concerns the design of bi- and trimetallic MOFs that can exhibit a synergic effect between different metal centers. Bimetallic MOFs have the extraordinary potentials mainly due to the abundance of active sites [28]. Trimesic acid (TMA) is a versatile organic linker commonly used to construct 3D metal–organic frameworks (MOFs). Its benzene ring structure and three carboxylic acid functional groups provide excellent opportunities for coordination with metal ions, leading to the formation of stable and diverse MOF architectures [40].

Multiple studies revealed that the inclusion of Copper (Cu) and Nickel (Ni) transition elements to MOFs leads to excellent sensing performance, as this increases the electrochemically active sites and sensitivity [41–43].

What distinguishes MOFs the most is the endless potential of enhancement of sensitivity by using different metals and organic linkers. There are 1D, 2D, or 3D MOFs depending on the number of metal ions coordinated to the organic linker. Although 3D MOFs are the most challenging regarding synthesis and characterization due to the increased complexity of incorporating several metals, they often function the best because they have more active sites, leading to more catalytic engagement thus a more efficient performance [22]. They show superior peroxidase-like activity, faster reaction speeds, and stronger affinity for substrates due to the synergistic electron transfer between the three metals [44]. Also, the structural stability of the MOF can be improved by the presence of several metals. This is especially helpful in extreme circumstances, including hot temperatures or acidic or basic environments [45].

White Analytical Chemistry (WAC) is the philosophy of sustainable development in analytical chemistry [46]. It is an idea that promotes the harmony and integration of analytical, ecological, and practical qualities to ensure the analytical method's sustainability. The greenness, blueness, and whiteness of the proposed electrochemical method was evaluated using four of the most recent assessment protocols.

The RGB model [47] represents sustainably in color white, which is a combination of mixing green (method safety), red (analytical performance), and blue(practical effectiveness).The method is sustainable when it succeeds to achieve the primary white color. The RGB model utilizes an excel sheet containing tables addressing different criteria, and each criterion has a point value ranging from 0 to 100, with 0 standing for the worst, and 100 being very well suited.

The Complex-GAPI is an extension to traditional GAPI, with an advantage of evaluating the method’s preprocess performed to the analytical method [48]. The preprocessing step may be a barrier for many scientists who are willing to apply a particular method, so this tool is very helpful in rating a much wider aspect of the analytical approach. The results are illustrated in a five-pentagram and one hexagonal figure. The five pentagrams represented sample collection and preparation, instrumentation, and method type, reagents and solvents: with additional hexagonal diagram representing the pre-analysis processes. Green represents excellent greenness, yellow represents medium impact on the environment, while red represents that this step is of high toxicity.

The AGREE metric is a very straightforward tool evaluating all of the 12 principles of Green analytical chemistry [49]. The output is a pictogram indicating the final score of 1.00, and shades of green.

The BAGI tool has an advantage of evaluating the method’s practicality, a crucial parameter that routine analysis laboratory encounters [50]. Results are generated as an asteroid pictogram, along with a numerical rating inside it. Colors in the pictogram consist of dark blue (referring to high compliance), blue (moderate compliance), and finally light blue (low compliance). For the method to be guaranteed practical, the final score should be higher than 60.

Using those four metrics simultaneously will provide comprehensive information about the strengths and weaknesses of the analytical procedures used, and a guide to future applications of this study.

The aim of this work is to develop a novel and sustainable bimetallic CuNi-MOF/CPE sensor depending on Trimesic acid, able to detect SLD at nano range. Various matrices were investigated like veterinary formulations, plasma, and animal products, and the capability of the sensor to reach the drugs MRLs was also studied.

## Experimental

### Materials and reagents

Polyvinyl pyrrolidone K30 (PVP), Trimesic acid (TMA), Cupper (II) sulphate, and Nickel (II) sulphate, graphite, DMF, and paraffin oil were purchased from Sigma-Aldrich. Phosphate-buffered saline (PBS) tablets, ethanol and acetonitrile of HPLC grade were purchased from Fisher-Scientific (Loughborough, UK). Double distillation (Agela Technologies Wilmington, USA) was used to prepare deionized water. Egg and milk samples were purchased from a local market. Female cow plasma was provided by Faculty of Veterinary Medicine-Cairo University. Sulphadimidine (SLD) with purity 100.06% ± 0.637 was generously provided by Pharma Swede Veterinary Company, Cairo Egypt.

Sulphadimidine Injection B.P.2011^®^ and Sulphastat® powder veterinary formulations were purchased from a local veterinary pharmacy.

### Instrumentation

For all voltametric measurements, a PC-controlled electrochemical analytical workstation (Metrohm Autolab potentiostat/galvanostat PGSTAT204) equipped with NOVA software for electrochemistry was used. Ag/AgCl was employed as the reference electrode, and Pt wire served as the counter electrode, while CPE was the working electrode. For the analysis of spiked animal plasma, milk, and egg samples, PR1MA™ Vortex Mixer VM- 3200 was used, and centrifugated by Centurian Centrifuge K1015 0.2 L (Chichester, UK). For pH adjustment, Jenway digital ion analyzer model 3300 along with Jenway pH glass electrode was utilized (Essex, UK) 3330 with Jenway pH glass electrode (Essex, UK). The MOFs’ structure was identified by XPS, which was collected on K-ALPHA (Themo Fisher Scientific, USA) with monochromatic X-ray Al K-alpha radiation − 10 to 1350 eV spot size 400 micro m at pressure 10^–9^ m mark with full spectrum pass energy 200 eV and at narrow spectrum 50 eV. The FTS- 3000 spectrometer was used to obtain the Fourier transform infrared spectrum (FT-IR) of the fabricated CuNi-MOFs. Scanning electron microscopy (SEM) image of CuNi-MOF was obtained by ZEISS EVO scanning electron microscope in Hamburg, Germany.

### Fabrication of bimetallic Cu/Ni- MOFs

The MOFs were prepared using solvothermal method [51]. Firstly, TMA (420.00 mg), NiSO_4_.6H_2_O (394.26 mg), CuSO_4_ (239.415 mg), and PVP (500.00 mg) were poured in DMF (30.00 mL), and 3 mL of 0.4 M NaOH was added dropwise while stirring for 20.0 min. The resulting colloidal suspension was then placed inside a 50-mL Teflon autoclave and exposed to a 12-h solvothermal reaction at 100 °C. The product was then allowed to cool to room temperature, then centrifuged for 14.0 min at 4000 rpm to produce the precipitate. This precipitation was then washed with ethanol and DMF and dried under vacuum for an hour at 65 °C.

### Fabrication of MOFs modified carbon paste electrode

The modified carbon paste electrode was prepared in different ratios as follows:

Ratio (9:1) or (10%MOF) was prepared with 90 mg graphite and 10 mg Cu/Ni- MOFs.

Ratio (19:1) or (5%MOF) was prepared with 95 mg graphite and 5 mg Cu/Ni- MOFs.

Ratio (99:1) or (1%MOF) was prepared with 99 mg graphite and 1 mg Cu/Ni- MOFs.

For each ratio, the weighed graphite was placed in a mortar and mixed with the corresponding amount of MOFs for at least 5.0 min using 10 µL paraffin oil. Each modified paste was filled inside the electrode body and a copper wire was utilized for electrode connection. For the unmodified CPE, 100 mg graphite was mixed with 10 µL paraffin oil but without adding MOFs. To renew the electrode surface, almost 3.00 mm of the old one was gently scrapped off with a tracing paper.

### Procedure

#### Standard solution

Stock standard solution of SLD 10^–2^ M was prepared by weighing 69.00 mg of pure SLD and transferring it into a 25-mL volumetric flask, then completing until mark with distilled water. Afterwards, 2.5 mL were taken into another 25-mL volumetric flask and completed with distilled water to obtain SLD concentration of 10^–3^M.

#### Operational conditions of electrochemical measurements

Regarding Cyclic Wave Voltammograms (CV), scanning was recorded over a range of − 0.1 to 1.2 V at room temperature. A CPE was used against the Ag/AgCl reference electrode, and a platinum wire as a counter electrode. As for Differential Pulse Voltammetry (DPV), measurements were carried out by scanning the potential over a range of − 0.4 to 0.5 V at scan rate 100 mV/s. The sample width, modulation amplitude, pulse width (modulation time), pulse period (interval), and quiet time were set to 17.0 ms, 40.0 mV,50.0 ms, 600.0 ms, and 5.0 s, respectively. These criteria were carefully selected to guarantee accuracy and dependability.

#### Construction of the calibration curve

Different SLD aliquots were taken from the stock solution (10^–2^ M) into a 25-mL volumetric flask and completed till mark using PBS pH 5.5, producing the final concentrations between the range (100 nM–100,000 nM). Differential Pulse Voltammetry was applied between − 0.4 and 0.5 V. To determine the calibration curve and regression equation, the current peak height for each sample was recorded and plotted against its corresponding concentration.

### Method validation

The cited method was validated according to ICH guidelines [52] in terms of accuracy, precision, quantification methods, linearity, and detection, to confirm its legibility for the anticipated usage. To examine linearity, six different concentrations of SLD were analyzed under the optimal electrochemical conditions. Accuracy was also investigated by obtaining the recovery percentage of three different concentrations. As for Intra-day and inter-day precision, they were also investigated and stated as %RSD.

### Method applications

#### Determination of SLD in dosage forms

##### Sulphadimidine Injection B.P.2011®

Each 1 mL contains 333 mg of SLD in the veterinary formulation. To prepare a concentration of 10^–2^ M, 0.83 mL were taken from the dosage form into a 100-mL volumetric flask and completed with distilled water. To prepare several concentrations, different volumes from this stock solution were transferred into a 25-mL volumetric flask and completed with PBS (pH = 5.5). The attained final concentrations were used to find the percentage recovery by substituting in the regression equation.

##### Sulphastat^®^ powder

Each gram of Sulphastat^***®***^ powder contains 359.3 mg of SLD. To prepare 10^–2^ M concentration, 0.77 mg were weighed and transferred into 100-mL volumetric flask then completed with distilled water. Dilutions and percentage recovery were obtained as mentioned above (2.6.1.1).

#### Determination of SLD in spiked animal plasma

For the stock solution, 69 mg of SLD were added to a 25-mL flask along with distilled water till mark. This was then used to produce several working solutions. Afterwards, 500 µL of each working solution was added to 500 µL plasma, and protein was precipitated using 1.5 mL acetonitrile. All mixtures were mixed using a vibrator vortex for 4 min at 3000 rpm, followed by centrifugation for 20 min at 4000 rpm. The supernatant was carefully transferred into a 25-mL flask and completed till mark with PBS (pH = 5.5). The obtained final concentrations were 1 × 10^5^, 8 × 10^4^, 5 × 10^4^, 1 × 10^4^, 1 × 10^3^, 1 × 10^2^ nM. Differential Pulse Voltammetry was applied in the range of − 0.5 to 0.5 V for the spiked samples. The current peak heights of the measured concentrations were used and correlated with the corresponding values to create a calibration curve. The percentage recovery of three replicates of three distinct concentrations was computed to assess the accuracy.

#### Determination of SLD in spiked animal products

##### Egg samples

One egg was transferred to a 50-mL beaker, and 50 mL distilled water was added and mixed until homogenized. Then 20.00 g of this mixture was weighed, and added into a 50-mL volumetric flask, and completed till mark with absolute ethanol. This mixture was vortexed for 5 min, and centrifugated for 30 min at 4000 rpm. The supernatant was collected carefully into separate falcon tubes.

Into a 25-mL flask, 500 µL of this supernatant, along with 500 µL of SLD stock solution prepared as mentioned (2.6.2), were added and completed with PBS (pH 5.5) till mark. Different volumes of stock solutions were used to prepare the desired concentrations, which were equivalent to 1 × 10^5^, 8 × 10^4^, 5 × 10^4^, 1 × 10^4^, 1 × 10^3^, 1 × 10^2^ nM, to determine the calibration curve and recovery percentages as mentioned above (2.6.2).

##### Milk samples

Aliquots of 500 µL skimmed milk, 1000 µL acetonitrile, and 500 µL of 10^–2^ M SLD concentration were added into 15-mL falcon tubes. The mixtures were vortexed for 4 minutes, and centrifugated for 20 minutes at 4000 rpm. The obtained final concentrations 1x10^5^, 8x10^4^, 5x10^4^, 1x10^4^, 1x10^3^, 1x10^2^ nM.The supernatant in each falcon tube was separated and added to a 25-mL volumetric flask and completed with PBS (pH 5.5). Calibration curve and recovery percentages were calculated as mentioned above (2.6.2).

## Results and discussion

This study utilized trimesic acid (TMA), a benzene- 1,3,5-tricarboxylic acid, as an organic linker to fabricate a 3D MOF, leveraging its unique chemical structure and functional properties. The three carboxylate groups symmetrically positioned around the benzene ring allow for multidentate coordination, enabling the formation of highly stable, extended 3D structures with significant porosity and surface area. These structural attributes are particularly advantageous for the differential pulse voltammetry (DPV) assay of sulfadimidine, as they enable effective adsorption of the drug molecules, enhancing their concentration near the electrode surface and improving assay sensitivity. The benzene core provides hydrophobic character and chemical stability, while the carboxylate-metal bonds ensure thermal and mechanical stability, allowing the MOF to maintain its crystalline structure during DPV for reproducibility and durability.

When paired with nickel or copper metals, TMA-based MOFs exhibit excellent redox activity and electron transfer capabilities, facilitating the electrochemical detection of sulfadimidine and enhancing the peak current response. The functional groups in TMA and the coordination chemistry of the MOF provide specificity for sulfadimidine, reducing interference from complex matrices. Additionally, the electronic and structural properties of these MOFs can be tailored through synthesis parameters to optimize drug interaction and improve assay performance. The synergistic effects of TMA and the metal centers contribute to sharp, well-defined electrochemical signals, lowering detection limits and enabling precise and reliable drug quantification. Furthermore, TMA-based MOFs are relatively easy and cost-effective to synthesize, making them a practical choice for routine pharmaceutical analysis. While PVP was incorporated as a polymer to prevent aggregation and provided a larger surface area with more active sites to engage with SLD, it also played a pivotal role in stabilizing MOF particles, enhancing porosity, and improving the framework's electrochemical properties. Its polar functional groups increased affinity for sulfadimidine, complementing the selectivity provided by trimesic acid linkers, while its stabilization effects ensured consistent redox activity and mechanical integrity under assay conditions. Additionally, PVP facilitated controlled MOF synthesis, optimizing crystal size and shape for enhanced sensitivity and reduced interference in SLD detection.

As a tribasic acid, TMA owns six ligands, which linked Cu^2+^ and Ni^2+^ stoichiometrically in three sites 3 * 2 = 6,4 * 1.5 = 6 3:1.5:1.5. This is shown in SF.1.

**Fig. 2:**
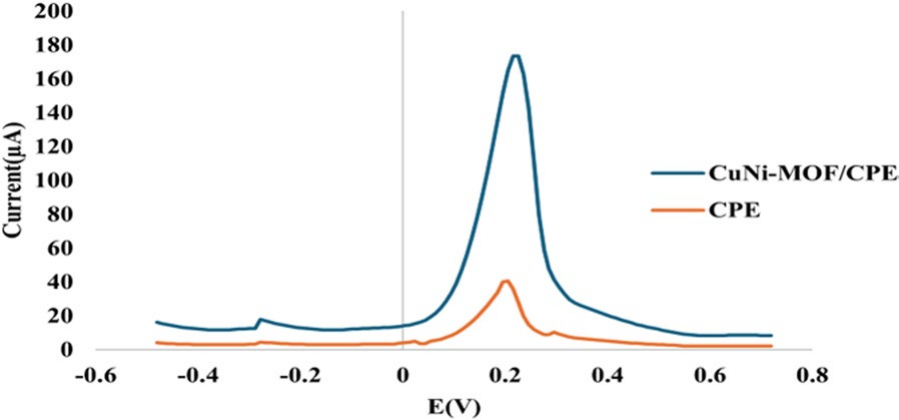
DPV of SLD using potassium ferro/ferricyanide solution (0.1 mM) in PBS buffer with CPE with and without CuNi-MOF

To explore the redox properties of Cu.Ni-MOF/CPE, differential pulse voltammetry (DPV) was conducted once on a plain CPE, and on the fabricated sensor as illustrated in Fig. 2. The modified sensor shows a better reduction–oxidation properties of ferro/ferricyanide solution, and more catalytic activity.

### Surface characterization

#### Scanning electron microscope (SEM)

The prepared CuNi-MOF’s morphology was investigated using SEM. Cu-MOFs usually have a well-defined octahedral structure, while Ni-MOF are usually acicular [43]. The structure of the prepared CuNi-MOF was in between as shown in SF.3(a), due to both Ni and Cu influence.

#### Energy dispersion X-ray (EDX)

Energy Dispersive X-ray Spectroscopy (EDX) is crucial in characterizing CuNi-MOFs for SLD determination, as it provides precise elemental analysis and confirms the successful incorporation of copper and nickel, which are vital for the MOF’s redox activity. EDX ensures the purity of the MOF by detecting impurities that could interfere with its performance and maps the distribution of metal centers to verify homogeneity, critical for consistent electrochemical detection. Additionally, it can confirm the presence of functional groups from modifications, such as polyvinyl pyrrolidone, which enhance the MOF’s functionality. By correlating metal concentrations to electrochemical performance and monitoring potential degradation or leaching, EDX ensures the stability and reliability of the MOF in repeated SLD assays. This examination was also conducted for elemental analysis. The mapping of the fabricated MOF indicated the presence of Cu, Ni, C, and O as seen in SF.3(b).

#### X-ray photoelectron spectroscopy (XPS)

The elements of CuNi-MOFs were further investigated by XPS technique conducted within the binding energy ranging from 0‒1400 eV. The indexed peaks of C, S, O, N, Ni, and Cu were clearly observed in the wide XPS spectrum analysis, strongly indicating the presence of the mentioned materials as shown in SF.3(c). The two prominent peaks at 934.55 and 954.31 eV are characteristic peaks of Cu 2p_3/2_ and Cu 2p_1/2_ states of Cu 2p respectively [53]. With a spin–orbit separation of 19.76 eV, the binding energies of these peaks could be interpretated by the + 2 oxidation of Cu. 942.0, and 950.1 eV were attributed to both Cu (2p) and Cu 2p_1/2_ [54]. Satellite peaks located at 961.1, 959.0, and 969.3 eV further confirmed the presence of CuO on the surface of the material [53]. Furthermore, peaks at 856.25 and 874.19 eV referring to Ni 2p_3/2_ and Ni 2p_1/2_ respectively validates the + 2 oxidation state of Ni [55]. Those observations clearly indicated electronic interactions between Cu and Ni in the fabricated MOF, which is very promising as this improves the electro-catalytic sensing of MOF.

There were also two characteristic peaks at 169.1 and 164.3 eV, referring to S 2p_1/2_ and S 2p_3/2_, indicating the presence of S^2‒^ state in transition metal sulfides, thus confirming the formation of CuS [56]. The spectrum of C provides information about the kind of carbon species found in the fabricated composite. Characteristic peaks at 285.8 and 289.2, and 292.38 eV refers to C=C, C=O and -C=O/-OH respectively. The high binding energy shoulder at 289.2 eV is attributed to the C=O bond found in the organic linker molecules [57].Moreover, the XPS spectrum of N(1 s) has two distinctive main peaks, 400.2 and 402.3 eV, related to pyridinic-N and pyrrolic-N respectively [57]. This observation referees to the MOF formation on the PVP polymer. Finally, The O 1 s spectrum portrayed peaks at 531.8 and 536.13 eV referring to Cu–O and C=O/–OH respectively. At 534.4 eV, the O 1 s peak overlaps with the existence of the Ni-O bond [45]. In summary, the XPS spectra corroborated the existence of Cu_2_O, CuO, Ni and NiO in the composites, which were in fine agreement with the results in literature.

#### Fourier transfer infrared spectrum (FT-IR)

The identification of surface functional groups and chemical bonds in the fabricated MOF was analyzed using infrared spectrum SF.3(d). Firstly, absorption peaks at 1114.86 cm^−1^ and 1315.45 cm^−1^ are observed, corresponding to the stretching vibrations of C=O and C-O, as well as the bending vibration of O-H, typical of the carboxylic acid coordination mode. Additionally, broad peaks in the range of 2735.06 cm^−1^ to 3448.72 cm^−1^ are attributed to the acidic OH groups of carboxylic acids. Peaks at 621 cm^−1^ and 671 cm^−1^ confirm the presence of Ni-O bonds, while the peak at 489 cm^−1^ is associated with the vibrational stretching of the Cu–S bond, confirming the existence of Ni and CuS in the prepared MOF. These peaks are linked to metal-oxygen-hydrogen bending vibrations (Ni-O, Cu-S, or Ni-Cu-O bonds) [58].

### Electrochemical characterization

Electrochemical impedance spectroscopy was conducted using 10 mM [Fe(CN)₆^3^⁻/Fe(CN)₆^4^⁻] redox probes. As shown in SF.4, the semicircles vary in size for each curve, indicating that the charge transfer resistance (Rct) of CuNi/MOF is lower than that of the bare CPE. The Rct values for the bare CPE and CuNi/MOF were 1.19 kΩ and 0.82 kΩ, respectively. This suggests that CuNi/MOF effectively coated the electrode surface and that the fabricated MOFs successfully reduce the Rct of the CPE, enhancing electron transport.

### Electrochemical performance of SLD on CuNi-MOF/CPE surface

#### Effect of buffer and pH

**Fig. 3:**
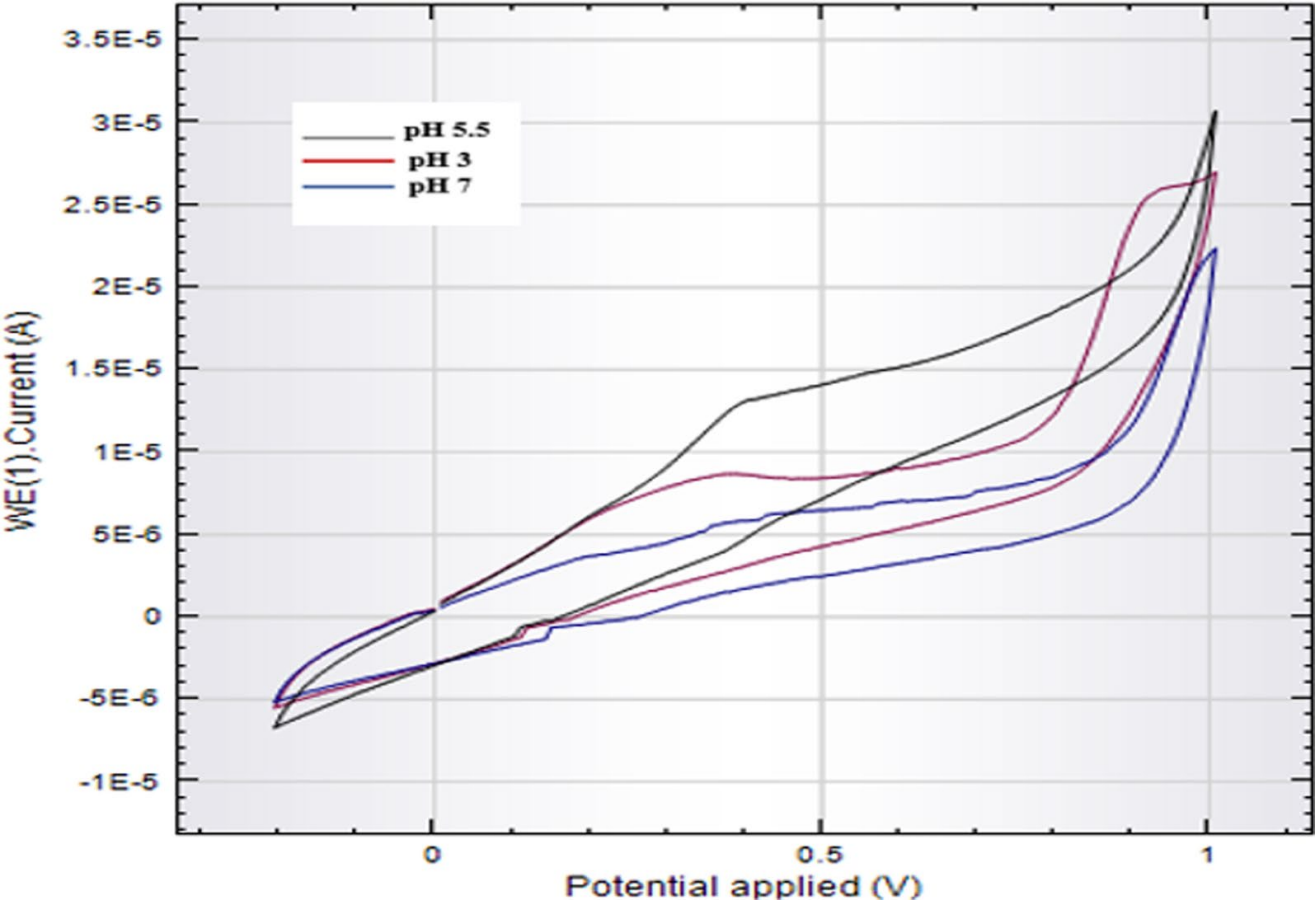
Effect of pH 3,5.5,7 on the cathodic peak current

Several conditions were investigated using CV to obtain the optimum sensing performance. As a start, different buffers were tried: Acetate buffer, phosphate buffer, and Britton-Robinson buffer, each from pH 2 to 9. The most sensitive response and highest peak potential of SLD was found with PBS at pH 5.5. This is logical, as SLD owns a pKa value of 7.1, so it has a positive charge at pH values lower than its pKa [59]. SF. 2 depicts the evolution of the peak current as a function of pH. It's evident that the peak current increases from pH 2.0 to 3.0, lowers from 3 to 4.5, then peaks between pH 5.0 and 5.5, and then begins to fall at higher pH levels—particularly over 7.0. It should be noted that at lower pH peaks were relatively high, and that at higher pH decline corresponds with the neutral form of SLD vanishes. An explanation for this could be that pH 5.5 is a compromise between thermodynamic and kinetic contributions to the SLD oxidation: at lower pH values, the presence of abundant H^+^-ions improves kinetics, but is thermodynamically unfavorable, whereas at higher pH thermodynamics is more favorable but the low concentration of H^+^-ions makes kinetics slower. Hence, pH 5.5 was selected for further experiments, which goes hand in hand with previously reported methods [60]. The effect of pH on the cathodic peak current was illustrated in Figure 3.

##### Effect of scanning rate

Scan rate is a key parameter when performing DPV, as it determines how quickly the potential is swept across the electrode. It is also related to diffusion layer thickness and peak shape. By varying the scan rate, researchers can gain insights into the kinetics and mechanisms of electrochemical reactions. Different scan rates from 10 to 100 mV/s using CV were investigated to understand the scan rate impact on the anodic peak oxidation of SLD. SF.5(a) illustrates the linear relationship between the current peak logarithm (Ip) and scan rate logarithm(Ʋ). The regression equation was found to be:

1 $${\text{log Ip}} = 0.5114{\text{ log}}\upsilon - { }5.8939{ }\left( {{\text{r}}^{2} = 0.9972} \right)$$

where the slope is higher than the theoretical value (0.50), indicating that the anodic reaction at the surface of the electrode is a diffusion-controlled process, owning some adsorption character. This is shown in SF.5(b) illustrated this regression equation.

Moreover, the following regression equation

2 $${\text{Ip}} = 1.1611{ }\upsilon \raise.5ex\hbox{$\scriptstyle 1$}\kern-.1em/ \kern-.15em\lower.25ex\hbox{$\scriptstyle 2$} + {\mathbf{0}}{\mathbf{.7171}} \left( {{\text{r}}^{2} { } = 0.9883} \right)$$

Indicated a directly proportional relationship between peak current (Ip) and scan rate square root(**Ʋ½).** This regression equation is illustrated in SF.5(c).

##### Effect of CuNi-MOFs ratio

To determine the optimum ratio for the best sensing performance, multiple ratios of CuNi-MOFs Ratios 9:1 (10%MOF), 19:1 (5%MOF) and 99:1 (1%MOF), graphite: CuNi-MOFs were prepared and investigated using DPV. SF.6 illustrated that ratio 19:1 graphite: CuNi-MOFs was the optimum ratio to achieve lowest concentration and stability. A reasonable explanation could be that ratio (99:1) had a very low amount of MOF which did not help in achieving high sensitivity. On the other hand, ratio (9:1) had the highest amount of MOF, and as mentioned in literature, a very well-known inevitable disadvantage in MOFs is the low adsorption capacity attributed to the microporosity [45]. Thus ratio 19:1 was the best ratio, overcoming all the mentioned obstacles.

### Suggested mechanism of SLD oxidation

Lavirons equation is used to determine the electrons involved in the reaction for an irreversible electrochemical process [61].

$${\text{E }} = {\text{ E}}0{ } + 2.303{\text{RT /}}\alpha {\text{nF}},{ }\left[ {{\text{log R at K}}0/\alpha {\text{nF}}} \right]{ } + { }2.303{\text{RT /}}\alpha {\text{nF }}\left( {\text{log v}} \right)$$

where, E is the applied potential, E^0^ is the formal standard potential, R is the resistance, K_0_​ is a constant related to the reaction, v is the scan rate, n is the number of electrons transferred in the reaction, F is Faraday's constant (96,485 C/mol), α is charge transfer coefficient which is usually 0.5 for an irreversible electron transfer and T is the temperature in Kelvin. For the proposed sensor, using slope (0.05144), obtained from potential against log scan rate linear plot, αn for SLD was calculated to be 1.032. It is assumed that irreversible electron transfer α is 0.5 [55], therefore “n” value was calculated to be 2.2, which suggests that the electrochemical reaction involves approximately 2 electrons, which is typical for many redox processes. This indicates that the reaction is likely to follow a two-electron transfer process, making it a relatively straightforward electrochemical process at the modified electrode. Sulfadimidine, a sulfonamide compound, can undergo oxidation at its aromatic or amino groups as shown in SF.7. The formation of 4-hydroxysulfadimidine involves hydroxylation of the aromatic ring, which requires Electron transfer from the aromatic system to the electrophilic attack by hydroxyl radicals or water molecules.

The MOF catalyst could facilitate this process by enhancing electron transfer and stabilizing intermediates. Copper centers are known to facilitate the generation of reactive oxygen species (e.g., hydroxyl radicals) in electrochemical reactions. These species are highly electrophilic and favor hydroxylation of aromatic systems. In addition, Nickel ions can enhance electrochemical activity by stabilizing intermediate oxidation states or synergizing with copper to promote the two-electron transfer mechanism required for hydroxylation. Ttrimesic acid provides a stable framework for the Cu/Ni centers and ensures proper coordination geometry, enhancing the accessibility of active sites for sulfadimidine oxidation.

At pH 5.5, the medium is slightly acidic, and the sulfadimidine molecule is partially protonated. This condition: Favors electrophilic attack on the aromatic ring, as the partial protonation enhances electron density at certain positions. Enhances the stability of hydroxylated products like 4-hydroxysulfadimidine by reducing competing side reactions, such as further oxidation or degradation.

The aromatic ring in sulfadimidine has electron-donating groups that direct electrophilic substitution preferentially to the para-position relative to the amino group. This makes the formation of 4-hydroxysulfadimidine more likely. Hydroxylation is favored over de-sulfonation or oxidative deamination due to the stability of the aromatic sulfonamide structure under these conditions. In addition to the catalytic properties of the MOF, which promote selective hydroxylation.

### Analytical performance of SLD

#### Calibration curve

The peak current of SLD was obtained by DPV. It was found that under optimal conditions, there was a good linear relation between the peak current and SLD concentration from 0.10 to 100.00 µM as shown in SF.8(a) and SF.8(b). The limit of detection was found to be 0.02 µM as shown in Table 1. The method proved its ability to detect very low concentrations of SLD, which would be very useful in applications and further investigations [62].

**Table 1:** Validation parameters obtained by the developed DPV method for the determination of SLD

Parameter	SLD
LOD (µM)	0.02
Linearity range(µM)	1 × 10^–4^–1 × 10^–7^
Slope	0.2413
Intercept	0.0679
r	0.9998
Accuracy (mean ± SD)	99.94 ± 0.74
Precision	
Inter-day (%RSD)^a^	0.51
Intraday (%RSD)^b^	0.29

^a^average of concentrations 20,30,60 µM of three replicate (n = 9) repeated on three successive days

^b^average of 20,30,50 µM of three replicates (n = 9) within the same day

### Method validation

To validate the proposed sensor, accuracy, precision, and reproducibility of the proposed sensor were examined. By examining 6 different concentrations of SLD and obtaining the recovery percentages, satisfying accuracy results of 99.94 ± 0.74 were obtained. The method was also proven precise according to the inter-day and intraday RSD% results of 0.51 and 0.29 respectively, which were less than 2. Those results were recorded in Table 1.

### Stability assessment

Throughout the study, the long-term stability of the sensor was investigated. The electrode was kept in the open air and measured every 6 days for 50 days to observe the changes in current peak values. At the end of the 50 days, the peak lost 8.97 % of its original value, as illustrated in SF.9. This is a very promising aspect of the proposed sensor, knowing that MOFs tend to lose stability quickly due lack the backbone to protect from structure deformation or aggregation [63].

### Application analysis

#### Detection of SLD in veterinary formulation

##### Sulphadimidine injection B.P.2011^®^

To evaluate the proposed sensor’s ability to perform adequately in practice, CuNi-MOF/CPE was utilized in detecting SLD in Sulphadimidine Injection. As very good recoveries of 99.83 %and 100.3% were obtained, the proposed sensor proved its ability to perform in SLD injections. Results were stated in Table 2.

**Table 2:** Results of SLD in veterinary formulations by the suggested DPV method

Drug trade name	Claimed (µM)	Found% ± SD*
Sulphadimidine Injection B.P.2011^®^ (1 mL contains 333 mg of SLD)	20	99.83 ± 0.68
	80	100.30 ± 0.51
Sulphastat^®^ Powder (1 gm contains 359.3 mg of SLD)	20	101.25 ± 0.74
	80	100.06 ± 0.62

^*^Found% = (found concentration/claimed concentration)* 100

##### Sulphastat^®^ powder

Another veterinary formulation found in the market was Sulphastat® Powder and also satisfying recovery percentages of 101.25% and 100.06% were obtained and stated in Table 2 by our proposed sensor, ensuring its success in detecting SLD in veterinary formulations.

#### Detection of SLD in animal plasma

This study also aimed at ensuring the detection of SLD by our fabricated sensor in plasma. To confirm this, a regression equation was obtained, and details recorded in Table 3, using plasma as a matrix, obtaining excellent detection, accuracy, and precision. Results of accuracy were 99.89 ± 0.93, inter-day and intraday precision %RSD obtained were 99.54% and 101.117% respectively, as shown in Table 3. The bright side is that the proposed method was able to detect SLD at its maximum residue limits (MRLs), which is 100 µg/L in plasma. This makes CuNi-MOF/CPE suitable for detection of SLD in real animal plasma and detecting if there are any violations to the MRL stated.

**Table 3:** Validation parameters obtained by the developed DPV method for the determination of SLD in spiked animal plasma, egg, and milk

Parameter	SLD spiked in:		
	Plasma	Egg	Milk
Slope	0.3122	0.9287	0.8019
Intercept	0.0647	0.0554	0.0566
r	0.9998	0.9999	0.9997

	Prepared concentration(µM)	Found concentration(µM)	Recovery %*
*Plasma*	20	19.90	99.52
	30	29.95	99.84
	50	49.27	98.54
	60	60.09	100.15
	80	80.18	100.23
	90	91.00	101.11
*Mean* ± *SD*			99.89 ± 0.93
*Egg*	5	5.08	101.55
	20	20.24	101.20
	30	30.17	100.56
	80	80.71	100.89
	50	49.84	99.69
	90	89.73	99.71
*Mean* ± *SD*			100.59 ± 0.77
*Milk*	20	19.40	97.01
	30	30.00	100.01
	50	50.32	100.64
	60	60.04	100.06
	80	79.47	99.34
	90	90.07	100.08
*Mean* ± *SD*			99.52 ± 0.46
Precision (RSD):			
Inter-day^a^	0.886	0.550	0.798
Intraday^b^	0.636	0.471	0.360

Accuracy (mean ± SD)

^*^ % Recovery = (found concentration/claimed concentration)* 100

^a^ average of concentrations 20,30,50 µM of three replicate(n = 9) repeated on three successive days

^b^ average of 20,30,50 µM of three replicates(n = 9) within the same day

#### Detection of SLD in animal products

It was also vital to confirm the applicability of the proposed sensor in detecting SLD in animal products commonly used such as egg and milk. A regression equation was obtained in both egg and milk matrix and detailed were shown in Table 3, and acceptable recovery percentages and precision %RSD were obtained. Regarding egg, accuracy obtained was 100.59 ± 0.77, while interday and intraday %RSD were 100.27 and 100.17 respectively as shown in Table 3. There is no MRL for egg, indicating that any detection of SLD is found violative. As for milk, accuracy obtained was 99.52 ± 0.46, while inter-day and intraday %RSD were 98.54 and 98.87 respectively. The proposed CuNi-MOF/CPE was able to detect SLD at milk’s (MRL), which is 25 µg/L. Those findings ensured that the fabricated sensor could easily analyze SLD in various animal products.

**Fig. 4:**
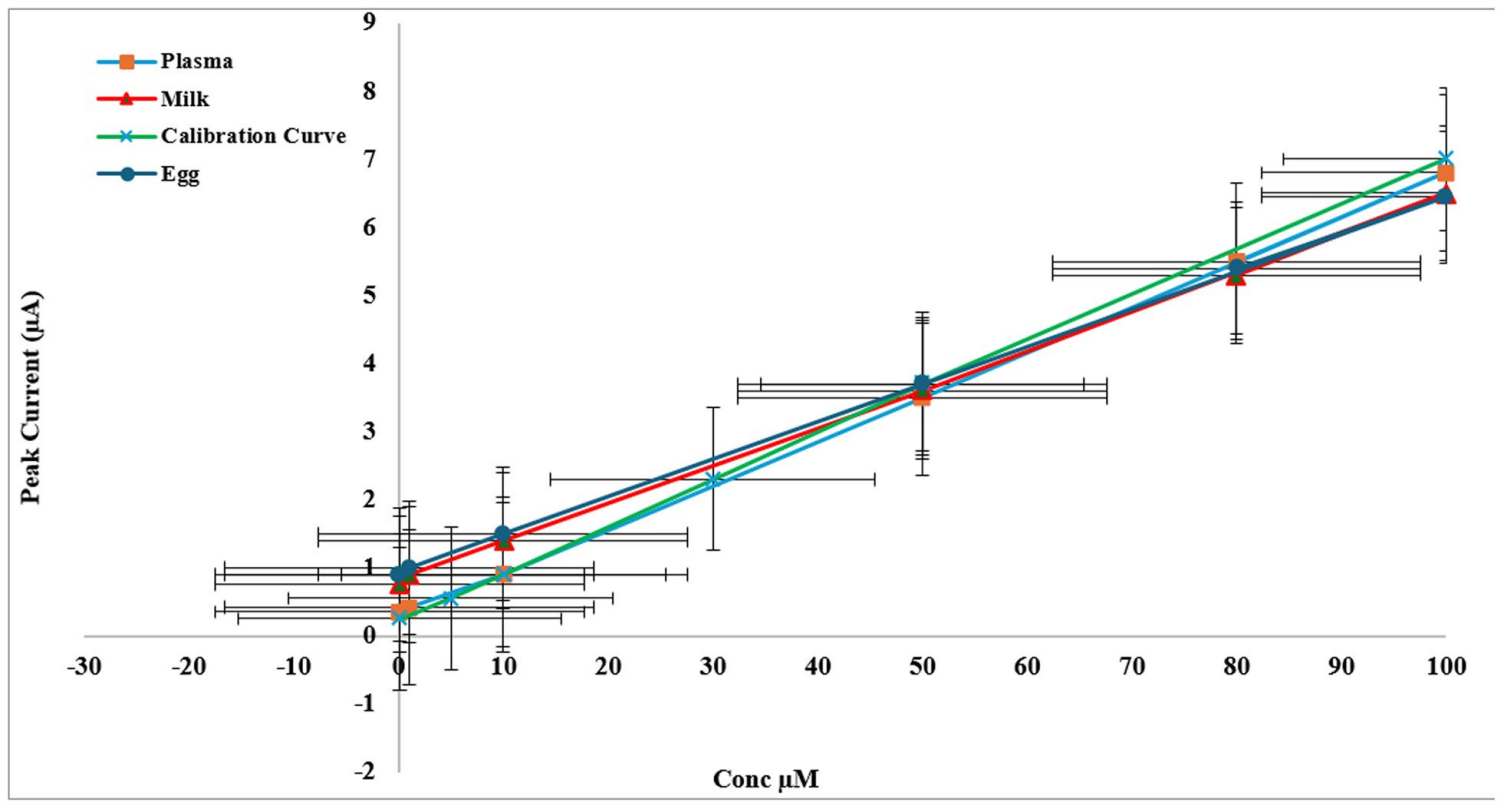
Calibration curves of peak current against SLD concentrations in PBS 5.5 spiked animal plasma, egg and milk samples

Figure 4 illustrates the calibration curves of all applications of SLD on CuNi-MOF/CPE.

The selectivity of the proposed bimetallic Cu/Ni-MOF/CPE was thoroughly evaluated to ensure its practical applicability in complex matrices such as veterinary formulations, animal plasma, milk, and eggs. The selectivity of the sensor was assessed by examining its response to potential interfering substances commonly found in these matrices, including other sulfonamides, antibiotics, and endogenous compounds such as proteins, lipids, and carbohydrates.

- Interference Studies: To evaluate the selectivity of the sensor, we spiked samples with common interfering substances, including sulfamethoxazole, sulfamethazine, and sulfaquinoxaline, which are structurally similar to SLD. The results demonstrated that the sensor exhibited a significantly higher response to SLD compared to other sulfonamides, with minimal cross-reactivity. This high selectivity can be attributed to the unique structural and electronic properties of the Cu/Ni-MOF, which provides specific binding sites for SLD due to the synergistic effect of the bimetallic centers and the TMA linker. The TMA linker, with its three carboxylate groups, creates a highly porous and stable framework that selectively adsorbs SLD molecules, while the Cu/Ni bimetallic centers enhance the redox activity, further improving the specificity of the sensor.
- Matrix Effects: The sensor’s performance was also tested in the presence of complex biological matrices, such as plasma, milk, and egg samples. The results indicated that the sensor maintained high selectivity for SLD even in the presence of high concentrations of proteins, lipids, and other endogenous compounds. This is particularly important for real-world applications, where the sensor must operate reliably in complex biological environments. The minimal interference observed can be attributed to the optimized pH conditions (pH 5.5) and the use of differential pulse voltammetry (DPV), which enhances the resolution of the SLD signal from background noise.
- Comparison with Other Methods: The selectivity of the proposed sensor was compared with other reported methods for SLD detection, such as chromatographic and spectrophotometric techniques. The Cu/Ni-MOF/CPE sensor demonstrated superior selectivity, particularly in complex matrices, where traditional methods often require extensive sample preparation and cleanup steps to achieve similar levels of selectivity.

The reproducibility of the sensor was rigorously evaluated to ensure its robustness and reliability for routine analysis. Reproducibility was assessed by analyzing multiple electrodes fabricated under the same conditions and by testing the same electrode over multiple measurement cycles.

- Electrode-to-Electrode Reproducibility: To evaluate the reproducibility of the sensor fabrication process, five different electrodes were prepared using the same protocol, and their responses to a standard SLD solution were measured. The relative standard deviation (RSD) of the peak current responses was found to be less than 2%, indicating excellent electrode-to-electrode reproducibility. This low variability confirms that the fabrication process is consistent and reliable, which is crucial for the mass production of sensors for real-world applications.
- Intra-day and Inter-day Reproducibility: The intra-day reproducibility was assessed by measuring the response of the same electrode to a standard SLD solution multiple times within a single day. The RSD for intra-day measurements was found to be 0.29%, demonstrating high repeatability. Similarly, the inter-day reproducibility was evaluated by measuring the response of the same electrode over five consecutive days. The RSD for inter-day measurements was 0.51%, indicating that the sensor maintains its performance over time without significant degradation. This high level of reproducibility is attributed to the stability of the Cu/Ni-MOF structure, which resists aggregation and maintains its electrochemical properties over extended periods.
- Long-term Stability: The long-term stability of the sensor was also investigated by storing the electrode in ambient conditions and measuring its response to SLD every six days over a period of 50 days. The sensor retained 91.03% of its initial response after 50 days, with only an 8.97% loss in sensitivity. This demonstrates the excellent long-term stability of the sensor, which is critical for its practical application in field settings where frequent recalibration may not be feasible.
- Real Sample Reproducibility: The reproducibility of the sensor was further validated by analyzing spiked samples of veterinary formulations, plasma, milk, and eggs. The recovery percentages for SLD in these matrices were consistently high, with RSD values below 2% for both intra-day and inter-day measurements. This confirms that the sensor can reliably detect SLD in real-world samples with high precision and accuracy.

### Statistical analysis

Upon comparing the results recorded in determination of SLD with the cited CuNi-MOF/CPE with the official methods [18], acceptable results were obtained. By using student-t test and F-test to compare the variance and the mean of both methods, no significant difference in obtained results was found. Details were shown in Table 4.

**Table 4:** Statistical comparison of the results obtained by the proposed and official methods for SLD analysis in its pure form

Parameter	Proposed method	Official method* [13]
Mean	100.07	99.59
SD	0.64	0.49
Variance	0.41	0.24
n	6.00	6.00
Student’s t-test (2.262)	0.316	
F value (6.26)	1.692	

*Official method includes dissolving 0.250 g SLD in 20 mL dil. HCl with addition of 3 g of potassium bromide, then cooling the mixture and titrating against 0.1 M NaNO_2_

#### Greenness and sustainability assessment

To ensure sustainability of the proposed method, the greenness, blueness, and whiteness of the proposed method was evaluated using four of the most recent assessment protocols; The RGB model was first created giving green, blue and white results. Then, each color was further assessed with a special evaluation tool to ensure alignment with RGB model results, and that different assessment techniques led to the same outcomes. Using those four metrics simultaneously helped completing each other’s drawbacks and provided comprehensive information about the strengths and weaknesses of the analytical procedures used. Details of each evaluation tool are described below.

#### The RGB model

**Fig. 5:**
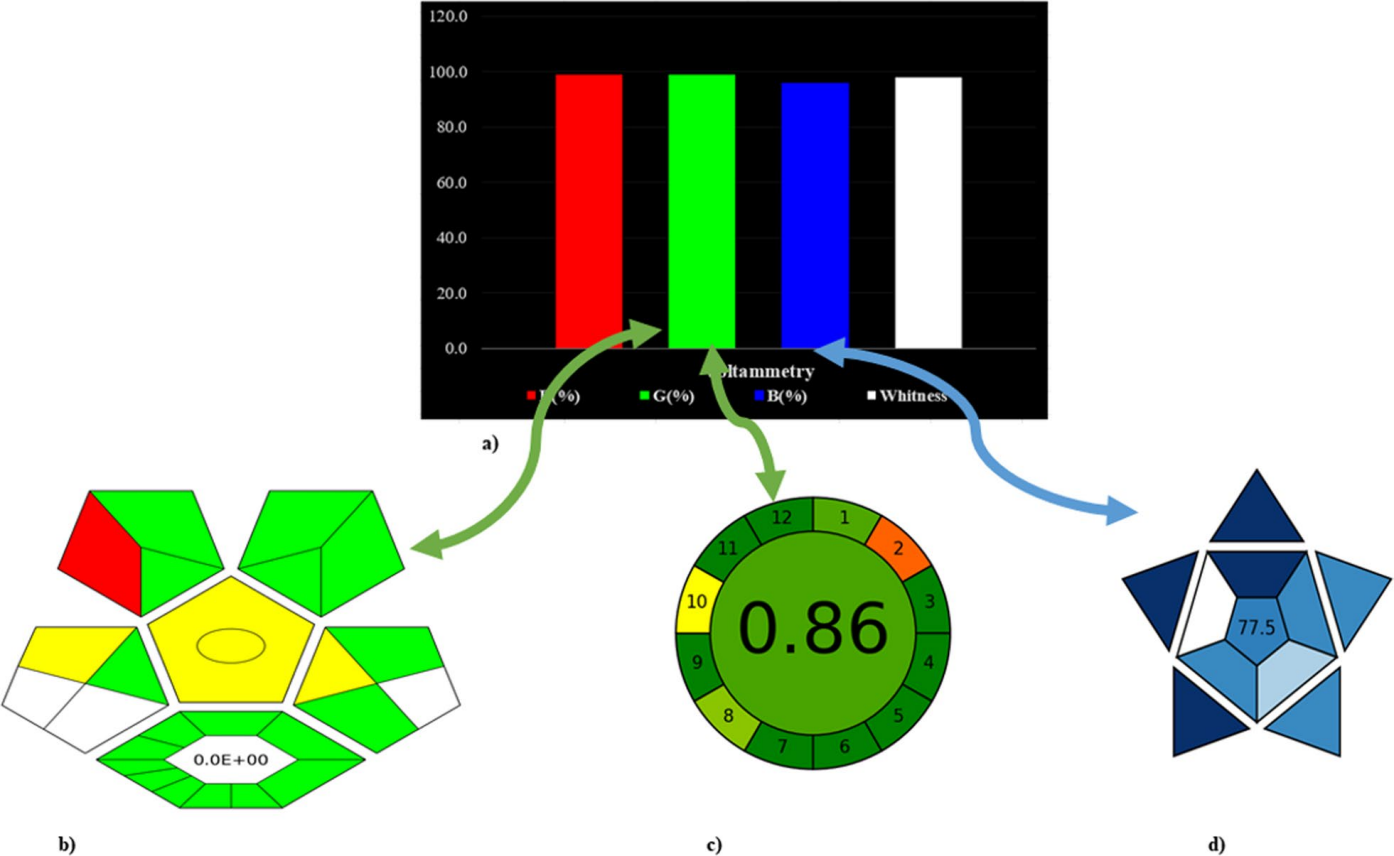
Sustainability Comprehensive assessment using **a** RGB model, **b** Complex GAPI, **c** AGREE, and **d** BAGI

The value of R (%), G (%), and B (%), while the overall result (whiteness%) of the proposed method is given in Fig. 5a

In the red category, precision, accuracy, LOD, and scope are evaluated. Our method showed an adequate score of 99%, referring to reliance and excellent sensitivity of the proposed sensor.

In the green category, toxicity of the reagents, amount of waste, energy consumption, and safety of the method was investigated. Based on the given data of the proposed method, a score of 98.8% was assigned by the RGB model, indicating greenness of the proposed sensor.

Finally in the blue category, practicality is inspected by assigning scores to time effectiveness, requirements, and overall simplicity. The cited method gave a score of 95.5%, referring to ease of application and functionality of the developed sensing system.

The method gave a superior white profile, mainly due to the safer solvents, low energy consumption, and easiness of applicability of the cited sensor. The electrode also can be renewed as it is mentioned in Sect."Fabrication of MOFs modified carbon paste electrode.", by gently scrapping almost 3.00 mm of the old one off with a tracing paper. To delve more and authenticate the scores, results of preprocessing, greenness and blueness were further confirmed by Complex GAPI, AGREE, and BAGI. All those tools have their own software to ensure ease of the process.

#### Complementary green analytical procedure index (complexGAPI) Vs. GREEN principle of RGB model

Regarding our proposed sensor, a red shade was given to solvent safety profile, yellow shades were assigned to method preparation (method requires fabrication of the working electrode by simple mixing of the graphite powder with the MOF) and storage. Additionally, there was no shade assigned to preservation, purification, transport, and waste as there was none of those. As there was nearly no waste produced, the method’s yield was almost 100% yield (E-factor = 0). The Complex Gapi figure showed in Figure 5(b) had green dominancy indicating the method greenness and preprocessing is superior.

#### Analytical GREEnness calculator (AGREE) Vs. GREEN principle of RGB model

Upon evaluating our proposed sensing system, the least score or red shade was given to the source of reagents as some of them like Trimesic acid had no safer substitutes. Light yellow shades contributed to storage as the electrode has to be stored under normal conditions, type of method as there were simple procedures were done like mixing and preparation of the MOF, and finally the energy amount, Other than that, all other aspects were given a high score, or dark green shade. Figure 5(c) illustrates an adequate total score of 0.86 was achieved, confirming the excellent greenness profile scores of the RGB model.

#### Blueness applicability grade index (BAGI) Vs. blue principle of RGB model

The proposed CuNi-MOF/CPE had light blue shade in the number of analytes simultaneously treated. That is because the 12 principles of GAC stated that it is better to determine more than one analyte at a time. The analytical technique has a blue shade, because it is neither sophisticated and tedious, nor very simple operation. Reconcentration requirements, degree if automation, and amount of sample also received a blue shade. Other than that, all other aspects received a dark blue shade. The method gave a total score of 77.5, referring to the sensor as easily applicable, which corelates with the blue region scores of the RGB model. The BAGI figure is shown in Figure 5d.

Several methods have been mentioned in literature for the electrochemical detection of SLD and the most recent were mentioned in Table 5. Carbon cloth was modified with metal-organic framework via a hydrothermal reaction with linearity range of 2.5–4.5 µg/L [64]. Also, home-made flexible integrated porous graphene (LIPG) modified with boron nitride have been described with linearity range 0.5 to 362.5 µM [65]. Another MOF consisting of lewis base was also fabricated to determine SLD [66]. Worth noting that the developed method gave more sensitive linearity and utilized greener and more sustainable steps.

**Table 5:** Previously described electrochemical methods for SLD determination

Method	Linearity Range
1. Carbon cloth (CC) modified with metal–organic frameworks (MOFs) [64]	2.5–4.5 µg/L
2. Home-made flexible integrated three-electrode of laser-induced porous graphene (LIPG) modified with boron nitrite [65]	0.5 to 362.5 µM
3. Lewis acid sites in MOFs to degrade SLD [66]	–

## Conclusion

This work succeeded in fabricating a bimetallic NiCu-MOF/CPE for the sensitive detection of SLD for the first time. Benzene- 1,3,5-tricarboxylic acid or Trimesic acid, is a tribasic acid that was selected as an organic linker to hydrothermally construct a bimetallic Cu/Ni MOFs, and was used to modify carbon paste electrode (CPE) for the electrochemical detection of SLD. The MOF was characterized using optical and electrical methods and proved to have superior structure. Several parameters have been investigated to decide the optimum condition which produced the best sensitivity and selectivity of the developed sensor. Excellent catalytic activity and electrochemical performance was achieved upon detection of SLD, due to the porous structure of MOFs, synergistic effect of Ni and Cu, and having multiple bonding cites due to the bimetallic nature of the MOF. The linearity range was able to detect in nanogram levels. Upon application to the veterinary formulations, adequate recoveries were achieved. Moreover, the linearity range succeeded in covering the MRLs of multiple drug matrices, like plasma, milk, and egg, which is promising in controlling antibiotic usage in animal warehouses. The method’s environmental impact was comprehensively studied by the RGB model, and compared to AGREE, Complex GAPI, and BAGI show excellent whiteness, greenness, and blueness profiles. This novel approach is a step towards achieving a very sensitive and simple method in detecting and controlling SLD usage in animals. A very promising fact is that modified MOF sensors can be incorporated into small portable systems for SLD detection on-site. This opens the doors for point-of-care diagnostics because patient care depends on the quick and accurate determination of SLD levels.

## Supplementary Information

Supplementary material 1

## Data Availability

No datasets were generated or analysed during the current study.
