# Supplementary material for: Introducing bimetallic MOF-based electrochemical sensor for voltametric nanogram determination of sulfadimidine: various applications and a comprehensive sustainability assessment
Source: BMC Chem. 2025 Apr 18;19(1):101. doi: 10.1186/s13065-025-01465-7 (PMC12008860; doi:10.1186/s13065-025-01465-7)
Supplement: Supplementary file 1 — Supplementary material 1 [file 13065_2025_1465_MOESM1_ESM.docx]

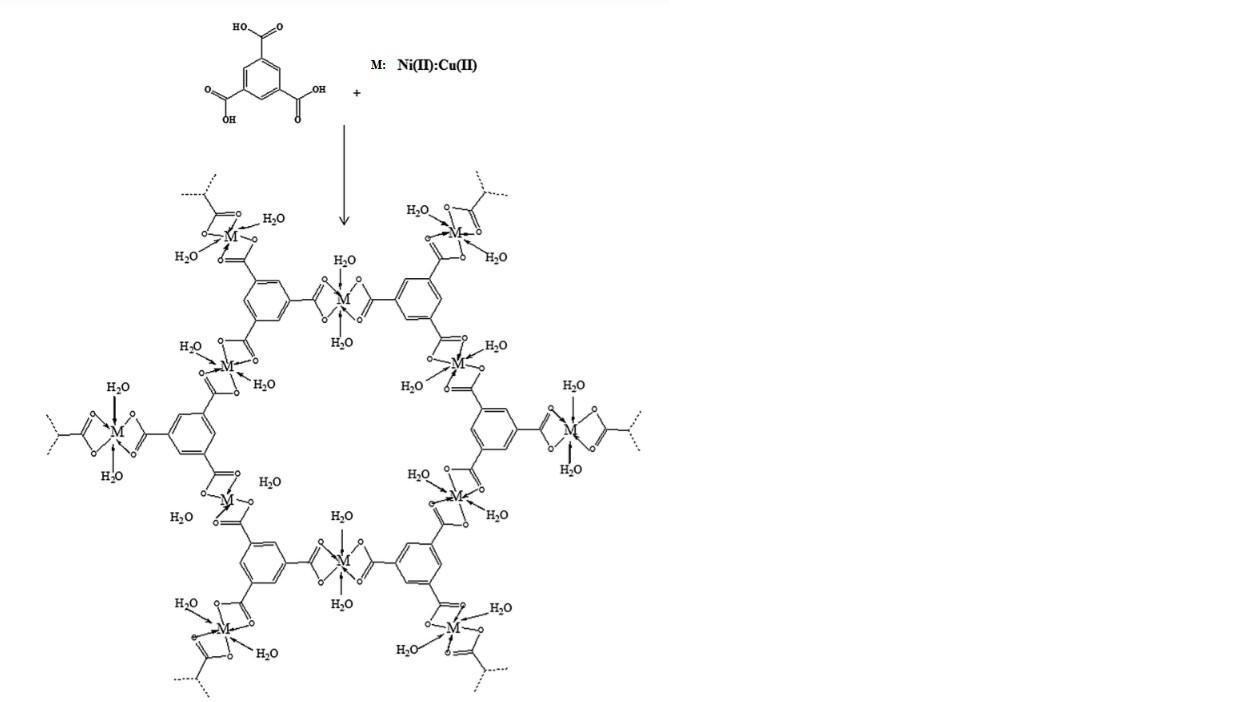


**SF. 1. Scheme illustrating Trimesic acid owning six ligands where Cu^2+^ and Ni^2+^ are linked stoichiometrically in three sites 3*2=6 ,4*1.5=6 ( 3:1.5:1.5)**

**SF.2. Changes in peak current of SLD in PBS buffer pH range of 2-9**


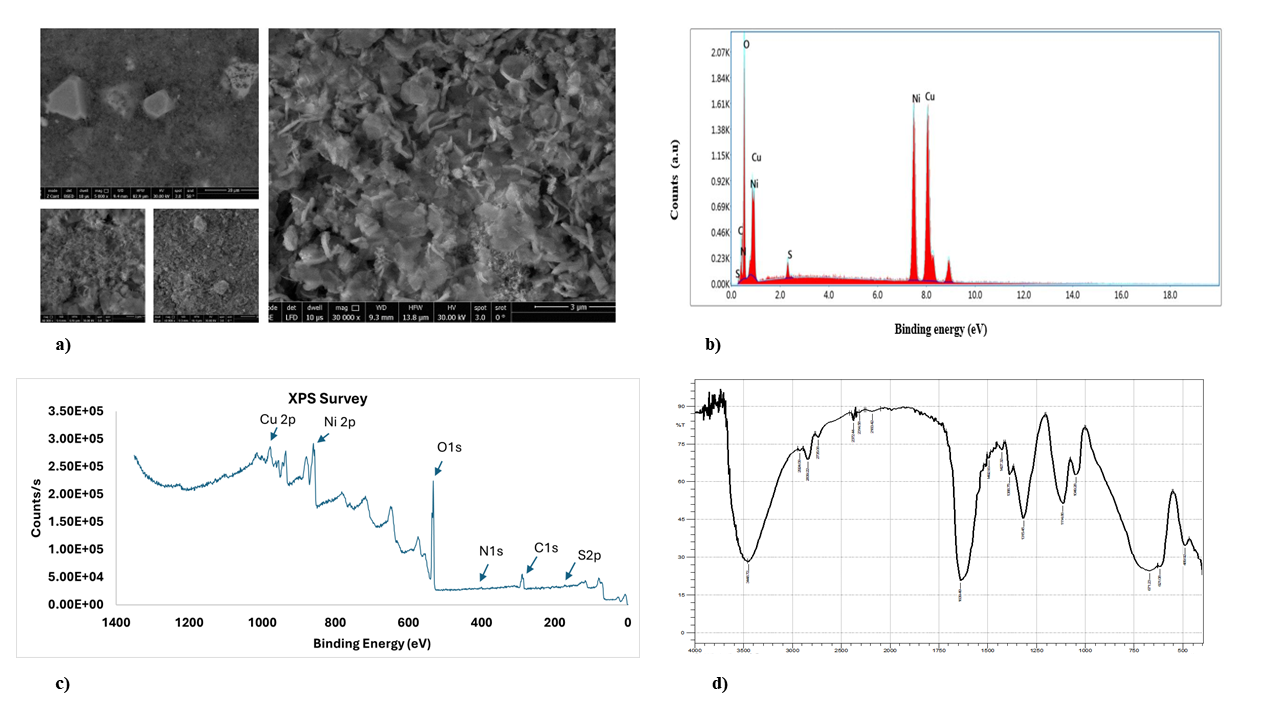
 **SF.3. Surface characterization of CuNi-MOF by:**

**a) Surface electron microscope (SEM) image**

**b) Energy dispersion X-ray (EDX) spectrum**

**c) XPS survey d) FT-IR spectrum**

**
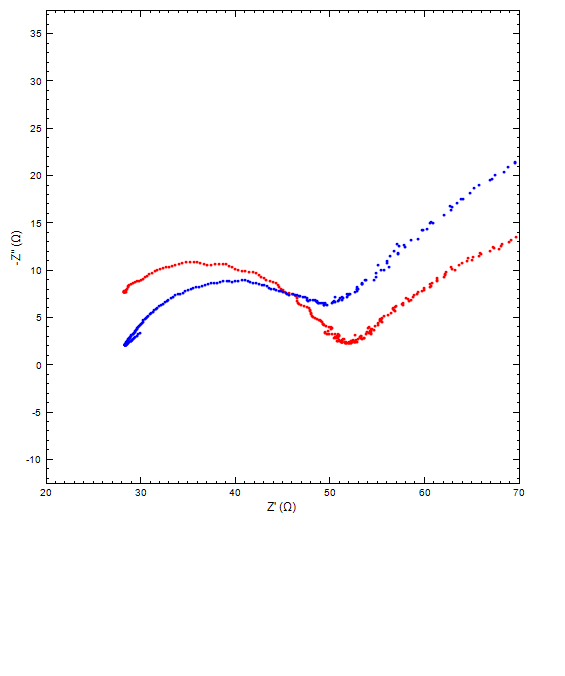
**

**SF.4. Electrochemical characterization of CuNi-MOFs by measuring EIS of CuNi-MOF/CPE (blue) and bare CPE (red) determined in 10 mM [Fe (CN)6^-3^/FE(CN)6^-4^]**


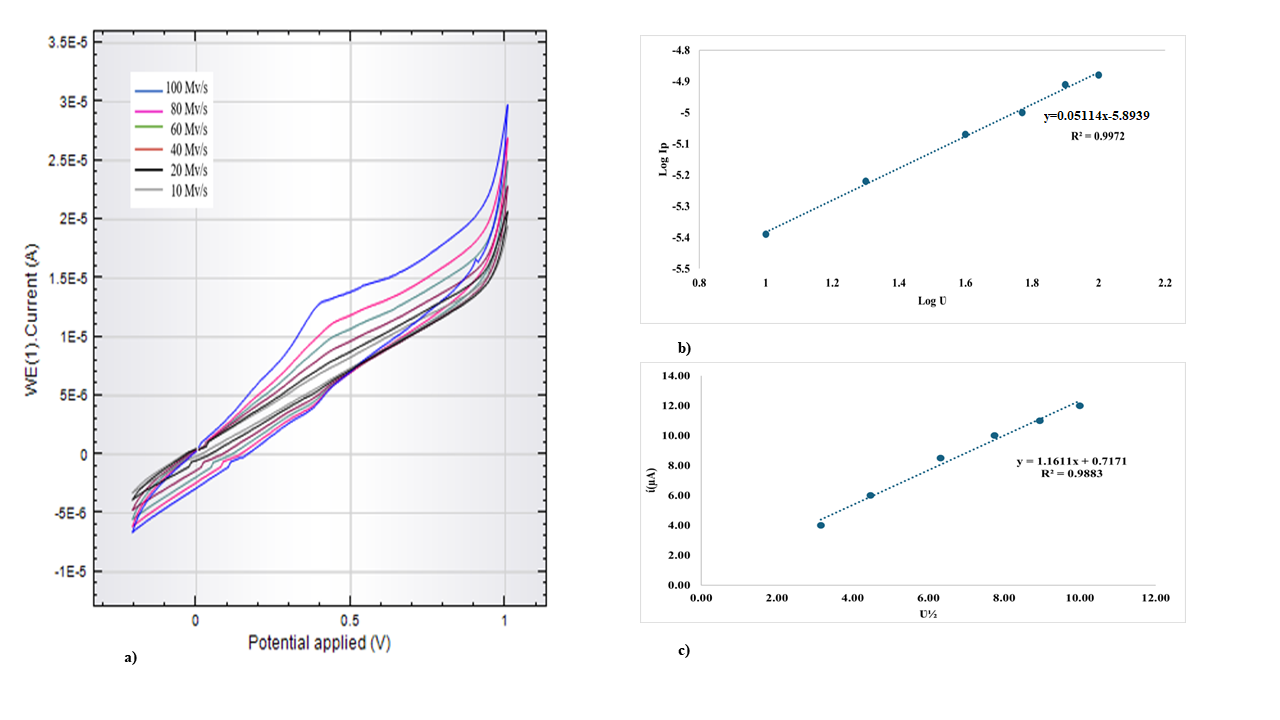
**SF. 5. a) Cyclic voltammograms of 10^-3^ M SLD in 10 mM PBS buffer (pH 5.5) on CuNi-MOF/CPE at scan rates (10,20,40,60,80, and 100 mV/s)**

**b) A plot of logarithm of anodic peak current (log Ip) as a function of logarithm of scan rate (logƲ) using CV.**

**c) A plot of the anodic peak current (Ip) as a function of scan rate (Ʋ^½^) using CV**

**
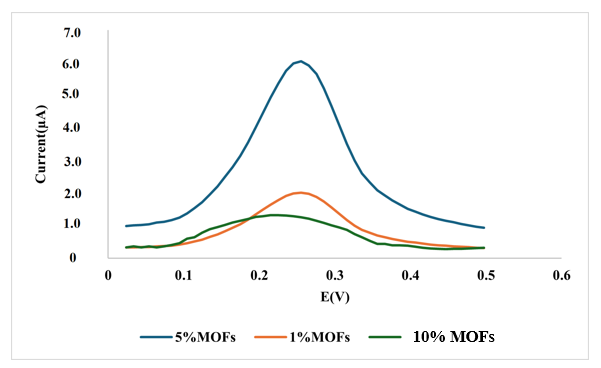
**

**SF.6. DPV of SLD on MOF ratios 1%, 5%, and 10 % using PBS buffer pH 5.5**


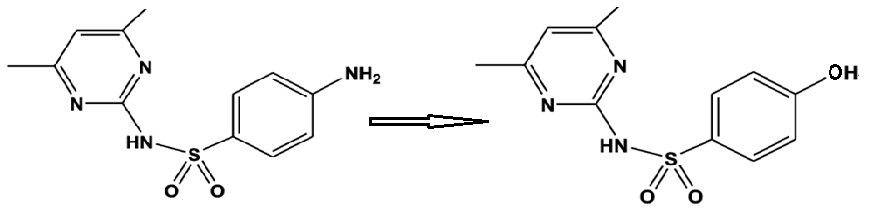


**SF.7. Scheme illustrating the possible electrochemical oxidation mechanism of SLD occurring at its aromatic or amino group.**


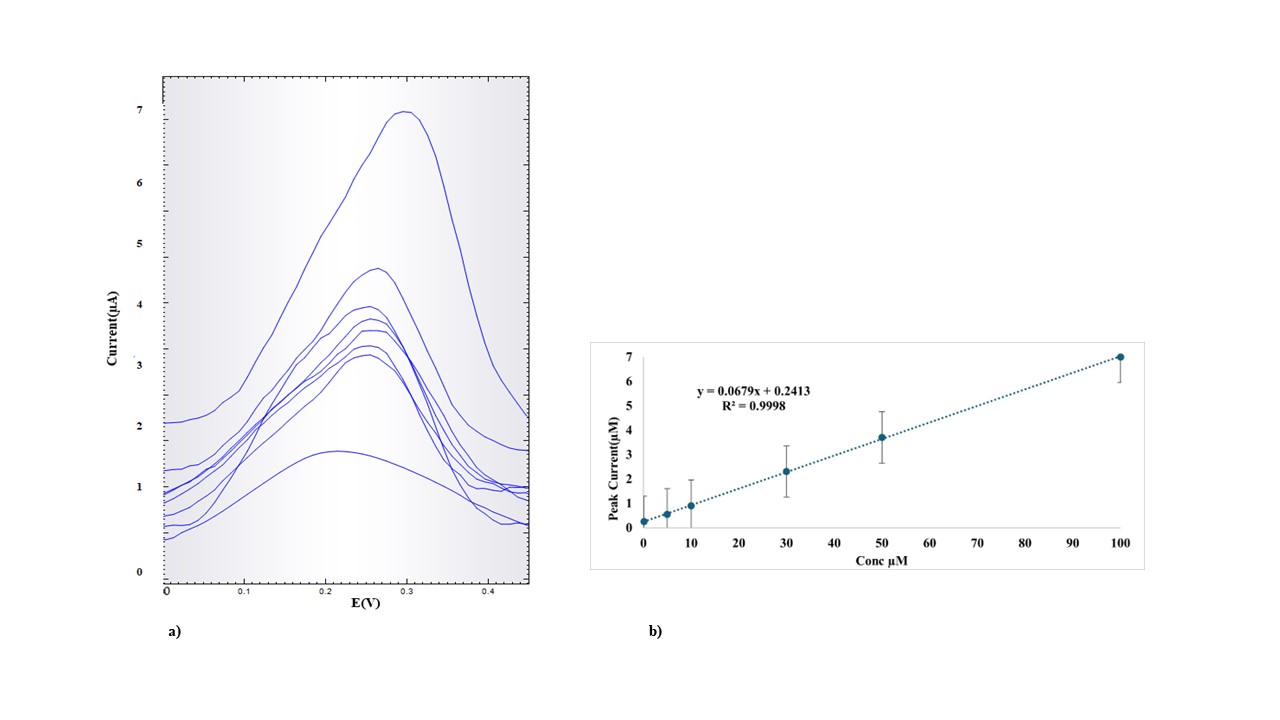


**SF.8. a) DPV of different concentrations (0.1,5,10,30,50,100µM) of SLD on CuNi-MOF/CPE using PBS buffer at pH 5.5. b) Calibration curve of peak current against SLD concentrations**


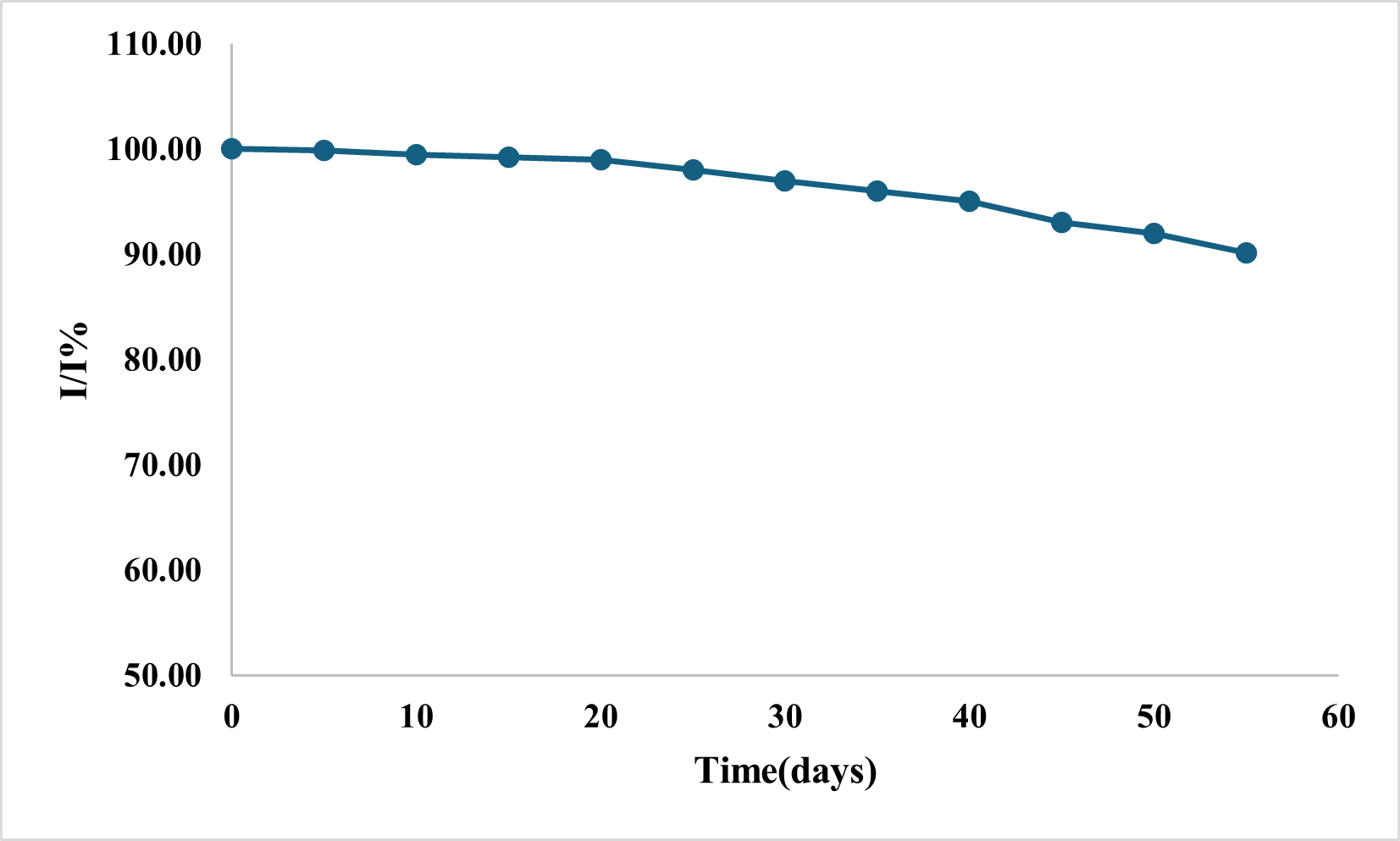


**SF.9. Long term stability (1-55 days) of CuNi-MOF/CPE**
